# Low Efficiency of Homology-Facilitated Illegitimate Recombination during Conjugation in *Escherichia coli*

**DOI:** 10.1371/journal.pone.0028876

**Published:** 2011-12-15

**Authors:** Jihane Amarir-Bouhram, Mélodie Goin, Marie-Agnès Petit

**Affiliations:** 1 INRA, UMR 1319, Micalis, Jouy en Josas, France; 2 AgroParisTech, UMR 1319, Micalis, Jouy en Josas, France; University of Minnesota, United States of America

## Abstract

Homology-facilitated illegitimate recombination has been described in three naturally competent bacterial species. It permits integration of small linear DNA molecules into the chromosome by homologous recombination at one end of the linear DNA substrate, and illegitimate recombination at the other end. We report that homology-facilitated illegitimate recombination also occurs in *Escherichia coli* during conjugation with small non-replicative plasmids, but at a low frequency of 3×10^−10^ per recipient cell. The fate of linear DNA in *E. coli* is either RecBCD-dependent degradation, or circularisation by ligation, and integration into the chromosome by single crossing-over. We also report that the observed single crossing-overs are *recA*-dependent, but essentially *recBCD*, and *recFOR* independent. This suggests that other, still unknown, proteins may act as mediator for the loading of RecA on DNA during single crossing-over recombination in *E. coli*.

## Introduction

**Figure 1:**
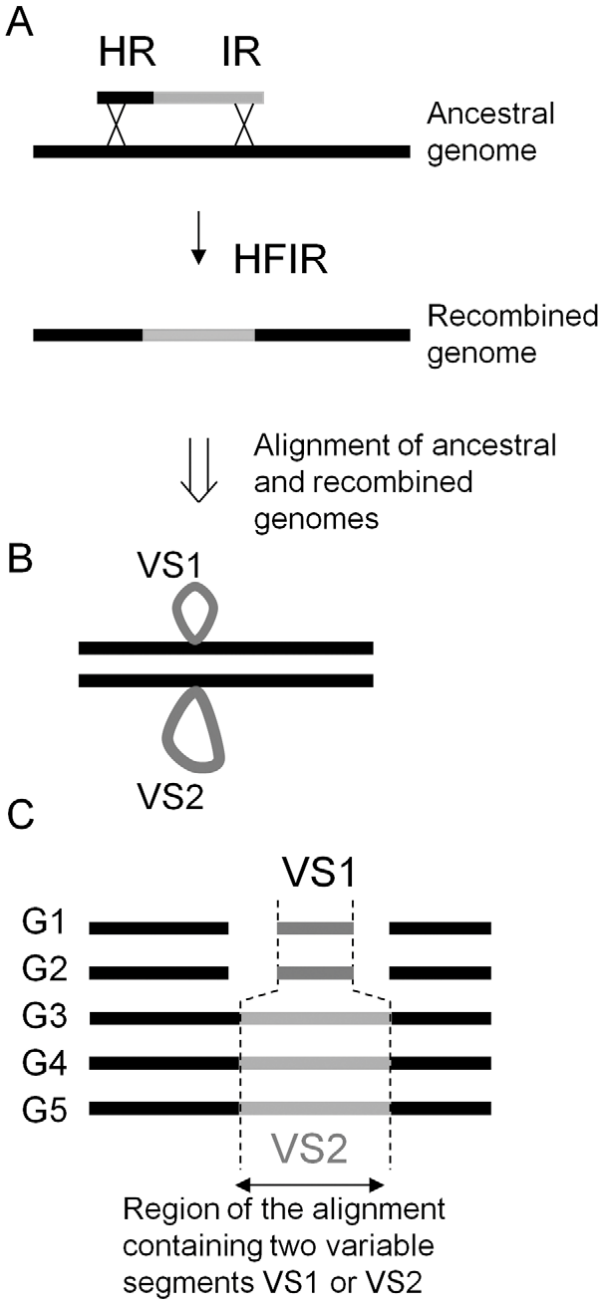
HFIR may account for the occurrence of insertions coupled with deletions, observed upon multiple genomes alignments. **A**. During Homology-Assisted Illegitimate Recombination (HFIR), a linear fragment sharing homology with the chromosome (black part) enters into the recipient chromosome by an atypical double crossing-over event, with homologous recombination (HR) at the left-end of the molecule, and illegitimate recombination (IR) at the other end. **B**. Alignment of the resulting recombinant with the ancestral genome will produce two small “variable regions” flanked by the backbone sequences, common to both strains. **C**. Diversification of strains produced by HFIR, leads to the detection of insertions (variable segment VS2 in genomes G3-G5) coupled with deletions (variable segment VS1 in genomes G1-G2) upon multiple genome alignments of various *E. coli* strains (here 5 strains).

Homology-facilitated illegitimate recombination (HFIR) is a hybrid recombination reaction whereby a linear DNA molecule integrates into the bacterial chromosome during natural transformation. Up to now, it has been reported in the three naturally competent bacterial species *Streptococcus pneumoniae* [1], *Acinetobacter baylyi* [2] and *Pseudomonas stutzeri* [3]. In HFIR, one end only of the linear incoming DNA molecule shares homology with the resident chromosome. Homologous recombination at this end is associated with an illegitimate recombination event within the non-homologous region of the linear fragment (see Figure 1A). In the recombination product, a deletion of a size similar to the inserted foreign DNA is generally observed (on average 1 kb insertions and 1 kb deletions). In most cases, a 3 to 10 bp micro-homology is found at the illegitimate recombination junction. Compared to the efficiency of homologous recombination, HFIR is 100 times less frequent in *S. pneumoniae* [1], 10^5^ times less frequent in *A. baylyi* [2], and 10^6^ times less frequent in *P. stutzeri*. HFIR is always more frequent than strictly illegitimate recombination, and in all cases the reaction is RecA-dependent. Moreover, in *A. baylyi*, HFIR efficiency is 20 fold increased in a *recJ* background [4]. RecJ is a 5′ to 3′ single-strand-specific DNA exonuclease [5], [6], which might degrade the 5′ single strand extremity required for the illegitimate recombination event in *A. baylyi*.

We recently performed a systematic analysis of the variable DNA segments found upon multiple alignments of *Escherichia coli* genomes, which revealed that a majority of them are small in size (less than 500 bp), and correspond to insertions coupled with deletions [7], as presented in Figure 1C. This situation could formally originate from HFIR events (Figure 1A and B). However, such recombination events have never been reported in *E. coli*, which is not naturally competent. We reasoned that other means of horizontal transfer might provide substrates similar to those used in HFIR in naturally competent cells. For instance, during DNA conjugation, single-stranded DNA is transferred, converted into double-stranded DNA, and may remain linear (and therefore a substrate of HFIR), if the DNA transfer is interrupted before completion.

We report the development of a genetic screen to detect HFIR events in *E. coli*, following DNA conjugation with small, non-replicative plasmids. We show that HFIR occurs once in every 3×10^10^ cells involved in conjugation, a frequency a million times lower than homologous recombination, and which might be too low to impact on genome evolution of *E. coli*. We also found evidence that linear DNA has two principal fates in *E. coli*: it is either degraded by RecBCD, or ligated and integrated into the chromosome by single crossing-over (SCO). This led us to enquire into the *E. coli* genetic requirements SCO between a non-replicative circular molecule and the bacterial chromosome. We show that SCO is clearly RecA-dependent, but only slightly dependent on the RecBCD and the RecF pathways. We also observed that SCO is inhibited by RuvABC, and is RecG-independent. Finally, SCO events were inhibited by a factor of 20 by the UvrD helicase. We conclude that the factors mediating the loading of RecA during SCO events, if they exist in *E. coli*, are not RecFOR nor RecBCD, and are unknown at present. Homologous DNA recombination, which has been studied in depth for years in *E. coli*, still has some aspects that remain to be elucidated.

## Materials and Methods

### Strains

All strains are listed Table 1. Strain β2163 [8] is a MG1655 derivative hosting RP4-2-Tc::Mu in the *glvB* gene of its chromosome [9]. RP4-2-Tc::Mu contains the transfer functions of the conjugative plasmid RP4, together with a kanamycine resistance gene, and a Mu-prophage inserted in its tetracycline resistance gene. In addition, this strain contains the *pir* gene and erythromycine resistance gene integrated at the *dap* locus, so that it requires 0.3 mM diaminopimelate for growth. An *Eco*K restriction and modification mutant derivative of strain β2163 was constructed (strain MAC1306), in which the two genes *hsdR hsdM* were replaced by a phleomycin resistance (phleo^R^) gene. Like strain β2163, MAC1306 contains the Mu prophage [9]. We verified that Mu did not interfere with the recombination assay (not shown). The single mutant Δ*hsdR*::FRT derivative of β2163 was also constructed (strain MAC1308), to use as a donor strain in conjugation experiments when the plasmid had to be protected from EcoK restriction in the recipient strain.

**Table 1:** Strains and plasmids used in this study.

Strains		
Name	Relevant genotype (all strains are MG1655 derivatives)	Source, construction
β2163	RP4-2-Tc::Mu Δ*dapA*:(Erm-pir)	[8]
MAC1306	RP4-2-Tc::Mu Δ*dapA*:(Erm-pir) Δ*hsdR*-*hsdM*::*phleo* ^R^	This work
MAC 1308	RP4-2-Tc::Mu Δ*dapA*:(Erm-pir) Δ*hsdR*::FRT	This work
JAC7	pIsceI (Amp^R^)	This work
JAC21	*recA306 srl::*Tn*10*	This work
MAC1348	*recA306 srl::*Tn*10* pIsceI (Amp^R^)	This work
MAC1394	*recB268*::Tn*10*	This work
MAC1397	*recB268*::Tn*10* pIsceI (Amp^R^)	This work
MAC1470	*recF400*:Tn*5*	This work
MAC1473	*recF400*:Tn*5* pIsceI (Amp^R^)	This work
MAC1497	*ruvA60*::Tn*10*	This work
MAC1357	*ruvA60*::Tn*10* pIsceI (Amp^R^)	This work
MAC1476	*recG*::*Tet6200*	This work
MAC1479	*recG::Tet6200* pIsceI (Amp^R^)	This work
MAC1354	*recD::*Tn*10*	This work
MAC1352	*recD*::Tn*10* pIsceI (Amp^R^)	This work
MAC1500	*recJ::PhleoR*	This work
MAC1350	*recJ::PhleoR* pIsceI (Amp^R^)	This work
MAC1503	*recO*::Tn*5*	This work
MAC1507	*recR:*:Tn*5*	This work
MAC1662	*uvrD::phleoR*	This work
MAC1511	*recF400::*Tn*5 recB268*::Tn*10*	This work
Plasmids		
pSW23T	oriV R6K γ, oriT RP4, Cm^R^	[8]
pIsceI	pUC, *cI 857*, pL:*I-sceI*	A. Lindner and M. Elez (Inserm U1001, Paris)
pJA1	pSW23T, cut cassette, Cm^R^	This work
pJA2	pJA1, *lacZ end*	This work
pJA3	pJA2, *lacZ beg*	This work

The MAC1306 or MAC1308 strains containing the various plasmids described below were used as a donor strain in the conjugation experiments. Recipient strains were either MG1655 (wild type strain) or MG1655 mutant derivatives listed in Table 1, containing plasmid pISceI. Mutant phenotypes were verified by UV sensitivity tests (for *recA*, *recB*, *recF*, *recB recF*, *recO*, *recR*, *recG*, *recJ* and *ruvA* mutants), T4gp2 sensitivity (*recD* allele), mutator phenotype (*uvrD*), and sensitivity to EcoK restriction of phage Lambda grown on non-modifying strains (*hsdR* and *hsdM* phenotypes).

### Plasmid constructions

To place DNA in a situation in which HFIR may occur, a system allowing plasmid delivery with high efficiency, and maintaining this plasmid linear in the recipient cell for as long as possible was designed. To reach high efficiency of plasmid entry, we set up a genetic system based on conjugation in *E. coli*, taking advantage of the mobilizable suicide plasmid pSW23T [8]. This plasmid carries the *oriT* transfer origin of RP4, and relies for conjugation on the RP4 conjugation genes provided in *trans* (RP4-2-Tc::Mu is chromosomally integrated in the donor strain). It also contains the vegetative replication origin of plasmid R6K, which relies on the Pir replication protein provided in *trans* in the donor strain. This system is conceived such that, once mobilized and introduced in a recipient strain, pSW23T no longer replicates and can only survive by recombining with the recipient chromosome.

**Figure 2:**
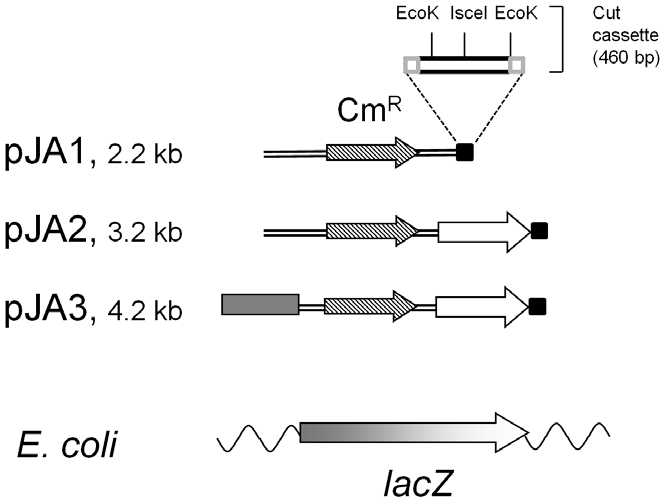
Maps of plasmids pJA1, pJA2 and pJA3 after linearization in the cut cassette. The 1 kb 5′ part of *lacZ* is shown with a grey box, and the 1 kb 3′ part of *lacZ* (lacZend) by a white arrow. The *cat* gene encoding resistance to chloramphenicol (Cm^R^) is shown as a stripped arrow. The cut cassette is indicated by a small black box, linearization of pJA plasmids *in vivo* might happen either at the IsceI recognition sequence, or between the two *Eco*K sites. The cut cassette map is shown in details, with two grey boxes indicating the Chi sites. Below the three plasmids, the *E. coli* chromosome with which plasmids might recombine is shown (wavy line) in the *lacZ* region (grey to white arrow).

Plasmid pSW23T was engineered further to maintain it in a linear form in the recipient strain, and thus facilitate HFIR detection. For this, a “cut cassette” composed of two *Eco*K restriction sites flanking an *I-sce*I restriction site was cloned into pSW23T, giving pJA1 (see Figure 2). *Eco*K is a type I restriction enzyme which loads DNA on the AAC(N6)GTGC sequence, then tracks along the DNA from its loading site, by pulling it from both sides [10]. Cutting occurs at random positions, upon collision with a second *Eco*K/DNA complex. Therefore, a minimum of two *Eco*K restriction sites are needed on a plasmid to provoke cutting. An internal segment of the *ycgN* gene of *Bacillus subtilis*, which contains two *Eco*K sites 450 bp apart was PCR amplified, using primers j1 and j2 (Table 2), containing at their 5′ extremities a Chi site oriented such that it protects pJA2 from RecBCD degradation, once linearised in the cut cassette. A linker composed of the *Isce*I cutting site (TAGGGATAACAGGGTAAT) and compatible ends (oligonucleotides j7 and j8, Table 2) was then ligated into the *ycgN* PCR fragment, between the *Cla*I and *Bcl*I sites. Finally, this synthetic cassette was cloned at the *Sma*I site of pSW23T, giving plasmid pJA1. The presence of all critical elements of this cassette was verified by DNA sequencing. This cassette allows the production, in the recipient strain, of a linear plasmid flanked by two Chi sites, properly oriented to protect linear DNA from RecBCD degradation.

**Table 2:** Sequence of the oligonucleotides used in this study.

Name	Sequence
j1	AACTGCAGCTGGTGGAAACACAGTGGTTCTGAAACC
j2	AATACCCGGGCTGGTGGCAGGTCCCATATAAACATCT
j3	ATACCTCCGCGGGCAACTCTGGCTCACAGTAC
j4	ATAGGATCCTTATTTTTGACACCAGACCA
j5	TTACTGCAGATGACCATGATTACGGATTC
j6	TTAGTCGACAAACCGACATCGCAGGCTTC
j7	CGATAGGGATAACAGGGTAAT
j8	GATCATTACCCTGTTATCCCTAT
j16	CCCAACCGCGTGGCACAACA
j17	GATTGAAAATGGTCTGCTGC
j18	AATGCCTCAAAATGTTCTTTACGA
j19	GCAACTCTGGCTCACAGTAC

In a further step, to provide the conjugative plasmid with homology to the recipient chromosome, a 1 kb segment corresponding to the 3′ terminal part of the *lacZ* ORF was amplified with primers j3 and j4, and cloned into pJA1 between the *Bam*HI and *Sac*II sites, giving pJA2 (Figure 2). Linearization of pJA2 at the cut cassette produces a molecule with 1 kb of homology to the *lacZ* chromosomal gene at one end (except for a few hundreds of terminal bp corresponding to the distance between the cutting site and the region of *lacZ* homology), whereas the other end has no homology with the recipient chromosome.

As a control for the efficiency of linearization, a pJA3 plasmid was derived from pJA2, containing the 5′ end of the *lacZ* ORF. This segment of *lacZ* was amplified with primers j5 and j6, and cloned between the *Pst*I and *Sal*I sites of pJA2. Linearization of pJA3 produces a molecule with 1 kb *lacZ* sequences at both ends, which are correctly oriented to integrate the plasmid by double crossing-over recombination in the *lacZ* gene in the *E. coli* chromosome, and produce a Lac^−^Cm^R^ ex-conjugant (Figure 2). If on the contrary pJA3 integrates into the chromosome as a circular form, by a single-crossing over recombination in either of the two *lacZ* segments present on pJA3, an intact *lacZ* ORF is restored, and a Lac^+^Cm^R^ phenotype is expected.

### Conjugations

Conjugations were performed between the MAC1306 or MAC1308 donor strains containing plasmid pJA1, pJA2 or pJA3, and various derivatives of the wild type strain MG1655 as recipient. The *Eco*K restriction system is active in MG1655. Plasmid pIsceI is a pUC derivative encoding the *cI* 857 thermosensitive repressor of bacteriophage Lambda, and the gene encoding the *Isce*I endonuclease cloned downstream of the Lambda pL promoter. Transcription of the endonuclease is induced by a shift from 37°C to 41°C for 1h30 prior to conjugation. Conjugations were done on filters at 41°C, unless otherwise stated, for two hours, starting from exponentially growing cells of the donor and recipient strains (OD_600_ of 0.3), and using a ratio of 2.5 recipient per donor cell. After conjugation, filters were taken, cells resuspended by vortexing in LB, and various dilutions of the mixtures were then plated at 37°C on LB rich medium devoid of diaminopimelate, to counterselect the donor cells, and to count recipient cells. Cm^R^ recombinants, both Lac^+^ and Lac^−^, were detected on LB plates supplemented with 20 µg/ml of chloramphenicol, 0.5 mM isopropyl-b-D-1-thiogalactopyranoside (IPTG) and 40 µg/ml of 5-bromo-4-chloro-3-indolyl-beta-D-galactopyranoside (X-gal).

## Results

### Experimental set-up to test HFIR

**Figure 3:**
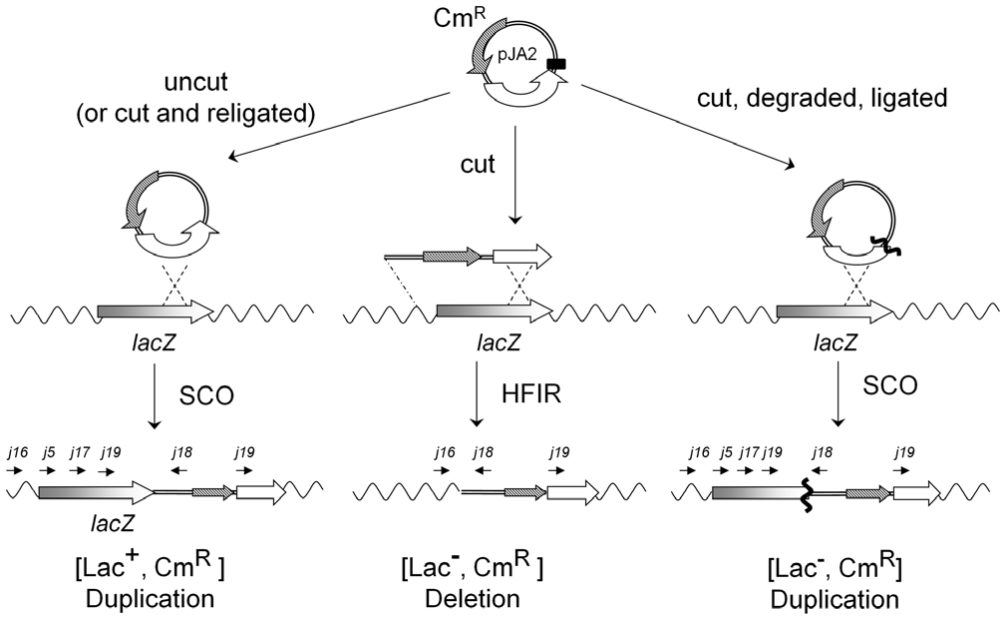
Inferred chromosomal recombination products obtained with plasmid pJA2. **Left part**: Single crossing-over (SCO) between circular pJA2 and the chromosome, as produced if pJA2 remains uncut (or if pJA2 is cut and resealed). The resulting exconjugants are Lac^+^ Cm^R^. **Central part**: HFIR between linear pJA2 and the chromosome. Exconjugants are Lac^−^ Cm^R^, and therefore distinguishable from the previous SCO events. **Right part**: If pJA2 is cut, degraded up into the *lacZ* sequence, and recircularized, a SCO between a circular, deleted (wavy bold line) pJA2 molecule and the chromosome will give rise to Lac^−^ Cm^R^ exconjugants. White arrow, 3′ part of the *lacZ* gene. Grey box, remaining part of the *lacZ* gene. Wavy lines, other sequences of the *E. coli* chromosome. Straight line, pJA2 plasmid sequences. Black box, “cut cassette”. Primers j18, j19, j17, j5, j16 complementary to the plasmid pJA2 and the chromosome, are shown above each chromosomal recombination product.

To detect HFIR during conjugation in *E. coli*, we used plasmid pJA2, and its two controls pJA1 and pJA3, presented in Figure 2. All plasmids encode a gene conferring resistance to chloramphenicol (Cm^R^), carry the *oriT* transfer origin of RP4, and the vegetative replication origin of plasmid R6K. Once mobilized and introduced in a recipient strain, such plasmids no longer replicate (nor conjugate), and can only survive by recombining with the recipient chromosome. To maintain the plasmid in a linear form in the recipient strain, the three plasmids contain a ‘cut cassette’, composed of sites cut by the two endonucleases EcoK and IsceI, framed by two Chi sites. Plasmid pJA2 has 1 kb of homology at the 3′end of *lacZ*, so that the linear pJA2 has homology to the chromosome at one end, and no homology at the other. With such a set up, HFIR is expected to produce Cm^R^ Lac^−^ exconjugants as represented Figure 3 (central part), which can easily be distinguished from the Cm^R^ Lac^+^ ex-conjugants formed by single crossing-over due to possible residual circular molecules (Figure 3, left part). Plasmid pJA3 is a control plasmid derived from pJA2 (see Figure 2), which permits measuring the efficiency of linearization. It contains a second 1 kb region of homology with *lacZ* (the 5′ end of the *lacZ* ORF). Finally, pJA1 has no homology to the chromosome.

### Estimation of the efficiency of linearization of the incoming plasmids after conjugation

All conjugative plasmids used in this study enter as single strand, linear DNA in the recipient cell. However, the plasmid recircularizes once entry is completed, and converts into double strand (ds) DNA. This conversion to ds DNA is probably rapid, as recombination of pJA2 into the chromosome is sensitive to EcoK and IsceI ds endonucleases (see below and Figure 3). In order to maintain the incoming ds DNA molecules linear, we made use of the resident restriction enzyme *Eco*K, and added an additional *Isce*I endonuclease provided by plasmid pIsceI. The efficiency of cutting by each system was estimated using pJA3. If all pJA3 molecules are kept linear, 100% of Lac^−^ recombinant clones are expected, because all Cm^R^ exconjugants will result from double crossing-over events (DCO) involving the two *lacZ* fragments present on the plasmid. If some of the molecules remain circular on the contrary, they will generate recombinants by single crossing-overs (SCO), which will reconstitute the full length *lacZ* ORF, and be Lac^+^. Finally, when no restriction is provided (*i.e.*, the donor strain is Modification^+^ for *Eco*K, and the recipient does not have plasmid pIsceI), the proportion of Lac^−^ clones will indicate how many DCO are generated starting from a circular substrate.

**Figure 4:**
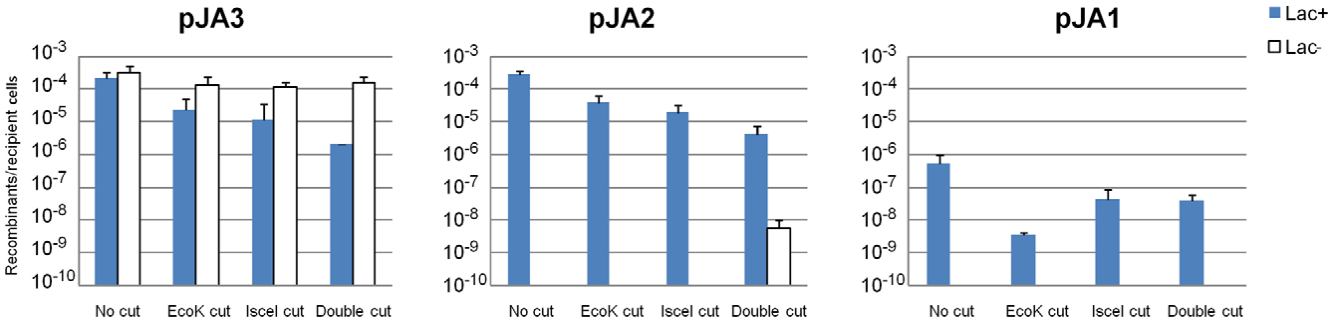
Efficiency of post-conjugative recombination with plasmids pJA3, pJA2 and pJA1. The Lac+ (blue bars) and Lac− (white bars, when present) Cm^R^ ex-conjugants reported to the viable recipient cells, are scored separately.

Conjugations with MAC1306 (EcoK Modification^−^) or MAC1308 (EcoK Modification^+^) donor cells containing pJA3 and recipient MG1655 wild-type cells, expressing or not expressing the IsceI site specific nuclease, were carried out on filters for 2 hours. Cells were then resuspended and appropriate dilutions were plated either on LB medium lacking DAP, to counterselect donor cells and count recipient cells only, or on the same plates supplemented with Xgal, IPTG and chloramphenicol, to count recombinant recipients and distinguish Lac^+^ from Lac^−^. Results are reported in Figure 4, left panel. We found that the background proportion of Lac^−^ clones obtained with pJA3 when no restriction is provided was 61%. Providing EcoK cutting or IsceI cutting increased the level of Lac^−^ clones to 86% and 91%, respectively. Finally, when both endonucleases were active, Lac^−^ clones were 98.7% of all ex-conjugants. We conclude that both cutting systems are efficient, even though 1.3% of exconjugants remain Lac+ when both systems are acting together, suggesting that a maximum of 1.3% of the plasmid molecules have remained circular (we suppose here that circular and linear molecules recombine with similar efficiencies).

### Recombination with linear pJA2 is decreased compared to linear pJA3

Next, conjugations with MAC1306 or MAC1308 donor cells containing pJA2 and recipient MG1655 wild-type cells, expressing or not the IsceI site-specific endonuclease, were carried out. Results are reported in Figure 4, middle panel. Efficiency of recombination with pJA2 decreased 10 fold when one endonuclease was active on the substrate, and 50 fold when both were active. The decrease in recombination yield for the linear molecules, compared to pJA3, is most likely due to the lack of homology at one end of the linear DNA. Despite the use of two endonuclease systems, *Eco*K and *I-sce*I, to linearise the incoming plasmids, a background ratio of 4×10^−6^ Lac^+^ Cm^R^ per recipient cells was obtained, which represents 2% of the recombinants obtained when the incoming plasmid is not cut. This is a ratio similar to that observed for pJA3 (1.3%), and corresponds to the inescapable background production of Lac+ clones resulting from single crossing-overs. We nevertheless detected 4×10^−9^ Lac^−^ Cm^R^ clones in the “double cut” context. No Lac^−^ Cm^R^ clones were scored when either one of the cutting system was used alone. We cannot rule out that this absence of Lac− is due to the technical difficulty of screening such clones among a wealth of Lac+ colonies. Alternatively, it may be that the sticky ends produced by each single restriction system reseal more easily when the two restriction enzymes are not combined.

A control experiment with plasmid pJA1 (Figure 4, right panel) sharing no homology with the *E. coli* chromosome showed a marked, 1000 decrease in the yield of Cm^R^ Lac^+^ clones compared to pJA2 or pJA3 when no cleavage was applied, as expected. These most likely were produced by illegitimate recombination. Interestingly, their yield was also reduced 10 to 100 fold upon cleavage. These clones were not analysed further.

### Detection of HFIR events with pJA2

We next proceeded to analyse the few Lac^−^Cm^R^ recipients produced with pJA2 when both cutting systems were active, as these were expected to correspond to HFIR events (see first section of the results). Lac^−^Cm^R^ recipients were found at a frequency of 5.8 (+/−4.0)×10^−9^ recombinants per recipient cells (60 Lac^−^ clones recovered over a total of 31 conjugation experiments, 0 to 7 Lac^−^ clones per experiment). All 60 Lac^−^ clones were analysed by PCR to test whether the integration of pJA2 into the chromosome had generated a deletion of part of *lacZ* as predicted for HFIR (Figure 3, middle). In most cases, unexpectedly, a duplication of part of *lacZ*, rather than a deletion, was observed (Figure 3, right part). The PCR test diagnostic for duplication use oligonucleotides j19 and j18, with j19 being placed at the limit of the *lacZ* end segment cloned into pJA2, and j18 facing j19, next to the Cm^R^ sequence (see Figure 3 lower panel for the positions of the oligonucleotides). In case of an HFIR event, these two oligonucleotides do not face each other (Fig. 3, middle) and no product is expected. In 56 of the 60 clones, a PCR product was found, whose size varied between 500 and 2 kb. Sequencing of 14 such clones confirmed the presence of a duplication of part of the *lacZ-end* homologous locus, with a size between 109 to 950 bp. We concluded that this category of Lac^−^ recombinants resulted from a single crossing-over between a pJA2 circular molecule lacking some of the *lacZ-end* fragment and the chromosome (see Figure 3, right part). Loss of some *lacZ* sequences in pJA2 could result from pre-existing plasmid deletion, or linearization by EcoK or IsceI followed by partial, RecBCD dependent degradation of *lacZ-end* and re-circularisation. The results below, showing that a *recD* mutation decreased the yield of Lac− clones, strongly suggests that the second alternative is more likely.

The remaining 4 of the 60 Cm^R^ Lac^−^ clones appeared to be the result of HFIR. To detect the new junction expected, a set of primers upstream from the *lacZ* 5′ locus (j17, j5 and j16, all 1 kb apart from each other, Figure 3, lower part) were tested in combination with j18, the pJA2-specific primer oriented towards the new junction. PCR products of different lengths were detected in all clones (one with the oligonucleotides combination j17-j18, three with j5-j18). Table 3 summarizes the deletion/integration length and the presence of homology at the junction for these clones. For 3 of the 4 clones, no homology was found at the new junction. We conclude that HFIR is detectable during conjugation in *E. coli*, albeit at a very low frequency. Based on the fact that 27 conjugations among 31 did not produce any HFIR clone, and using the first term of the Poisson distribution, we can estimate a frequency of HFIR of 3.5×10^−10^, which is 10^6^ fold below the frequency of homologous recombination events.

**Table 3:** Properties of the HFIR clones obtained.

Strain name	Size of *lacZ* deletion, upstream of *lacZ*-end	Size of the pJA2 insert	Homology at the junction	Sequence of the joint^a^
MAC1343	1081 bp	1747 bp	None	5′**ATGTCGGTTT**CCGCGAGGTG 3′lacZ 5′TATCGATACC**GTCGACGCCG** 3′pJA2
JAC233	1357 bp	1750 bp	None	5′**TTTGCCGACC**GCACGCCGCA 3′lacZ 5′GCGTTCGAAA**ATTCTCGTAC** 3′pJA2
JAC228	245 bp	1989 bp	None	5′**GCGCTGTACT**GGAGGCTGAA 3′lacZ 5′CTTATCGATA**CCGTCGACGC** 3′pJA2
JAC227	1385 bp	1974 bp	1 nt	5′**GCCACTCGCT**TTAATGATGA 3′lacZ 5′CGTACCAACT**TCTCTTGATC** 3′pJA2

a The joint is composed of the concatenation of the two bold sequences from each parent.

### SCO are nearly independent of RecBCD and RecFOR, and inhibited by RuvA

To better understand recombination in our assay, experiments were performed in various mutants. RecA is the central actor of all homologous recombination reactions, while RecBCD and RecFOR are mediator proteins facilitating access of RecA to single-stranded DNA. The RecBCD complex is specialised in promoting RecA loading at the extremity of linear ds DNA substrates, whereas RecFOR act on single-stranded gaps. The RuvABC complex resolves Holliday junctions into recombination products at the last step of recombination, and RecG is involved in a redundant pathway of resolution (for a review, see [11]). RecJ is a 5′ to 3′ single-strand exonuclease acting in the RecF pathway, and has been reported to prevent HFIR in *S. pneumoniae* [4], and UvrD displaces toxic RecA filaments [12].

Before testing recombination in strains permitting the linearization of pJA2, where several events are taking place (SCO and HFIR), we first examined the genetics of SCO, using donor and recipient strains that do not lead to pJA2 linearization. Efficiencies of recombination, measured as Cm^R^/Recipient cells, are reported in Table 4. Recombination was found to be *recA* dependent, but *recB*, *recD* and *recF* independent. Mutants for *recD*, *recO* and *recR* were not affected either. The double *recB recF* mutant yielded to a 2 fold reduction in recombination efficiency. Finally, recombination was increased by a factor of 17 in the *ruvA* mutant, and 22 in a *uvrD* mutant.

**Table 4:** Recombination frequencies with circular pJA2 in *E. coli* mutant derivatives.

MAC1308 Donor×Recipient strain (1)	Relevant genotype of recipient	Nb of experiments	Av. Cm^R^/recipient	Fold, relative to WT(2)
MG1655	WT	5	3.3 (+/−1.1)×10^−4^	1
JAC21	*recA*	4	2.7 (+/−2.3)×10^−7^	0.0006 **
MAC1394	*recB*	4	2.7 (+/−0.9)×10^−4^	0.58
MAC1354	*recD*	4	6.2 (+/−2.7)×10^−4^	1.9
MAC1470	*recF*	4	4.8 (+/−1.6)×10^−4^	1.45
MAC1503	*recO*	4	5.1 (+/−2.8)×10^−4^	1.5
MAC1507	*recR*	4	3.5 (+/−1.6)×10^−4^	1.04
MAC1350	*recJ*	4	4.0 (+/−2.0)×10^−4^	1.2
MAC1511	*recB recF*	4	1.8 (+/−0.5)×10^−4^	0.54 *
MAC1497	*ruvA*	4	5.6 (+/−4.4)×10^−3^	17 *
MAC1476	*recG*	4	2.7 (+/−0.8)×10^−4^	0.8
MAC1662	*uvrD*	4	7.2 (+/−2.9)×10^−3^	22 *

(1) Conjugations performed at 37°C.

(2) *Significant below 5%, ** Significant below1% with a Student test.

Intrigued by the limited effects of the *recB* and *recF* mutations on SCO during conjugation, we asked whether the efficiency of SCO during electroporation was also unaffected by these mutations. The same bacteria were prepared for electroporation, and transformed with pJA2. As a control for transformation efficiency, 1 ng of the replicative plasmid pACYC184 was used. Recombination yields were measured relative to the transformation efficiency. In the MG1655 wild type strain, a yield of 1.4×10^−4^ Cm^R^ recombinants per transformed cells was obtained. In the *recB* and *recF* derivatives, yields of 4.8 and 4.3×10^−5^ respectively were found, meaning once again that SCO recombination is barely affected (3 fold) by defects in the *recF* or *recB* pathways.

We finally measured recombination efficiencies in mutants with linear pJA2, i. e. under conditions of cleavage by both EcoK and IsceI. Both Lac^+^Cm^R^ (SCO products) and Lac^−^Cm^R^ (essentially pJA2 trimming, followed by SCO) are reported in Table 5. Compared to circular pJA2, similar effects of the *recA*, *ruvA* and *recJ* mutations were observed. A slight change was found for the *recB*, *recF* and *recG* mutations, as a small but significant 4 to 5 fold reduction in the yield of recombinants was observed, compared to the wild type situation. This means that with the linear pJA2 substrate, ∼1/5th of the recombinants produced by RecA occur in a RecB, RecF and RecG independent manner. The recombination assay with linear pJA2 could not be performed in *uvrD* mutant, due to the instability of plasmid pIsceI in this context, nor in the *recB recF* double mutant due to its low viability.

**Table 5:** Recombination frequencies with linear pJA2 in *E. coli* mutant derivatives.

MAC1306 Donor×Recipient strain	Relevant genotype of recipient	Nb of expe-riments	Av. LacZ^+^Cm^R^/recipient	Fold, relative to WT(1)	Frequency LacZ ^−^Cm^R^/recipient(2)	Fold, relative to WT	Proportion of HFIR events among LacZ^−^
JAC7	WT	31	4.2 (+/−3.4)×10^−6^	1	4.1×10^−9^	1	4/60
MAC1348	*recA*	12	1.3 (+/−0.8)×10^−8^	0.003 **	<8.7×10^−10^	<0.21	NA
MAC1397	*recB*	12	7.0 (+/−7.0)×10^−7^	0.17 **	<2.1×10^−9^	<0.26	NA
MAC1473	*recF*	12	1.1 (+/−0.6)×10^−6^	0.26 **	1.8×10^−9^	0.31	1/9
MAC1357	*ruvA*	12	2.5 (+/−2.8)×10^−5^	5.8 *	1.1×10^−8^	1.90	0/9
MAC1479	*recG*	12	7.5 (+/−6.3)×10^−7^	0.18 **	1.5×10^−9^	0.26	0/2
MAC1352	*recD*	15	1.8 (+/−1.0)×10^−5^	4.2 **	4.4×10^−10^	0.07	0/3
MAC1350	*recJ*	12	3.7 (+/−2.6)×10^−6^	0.87	6.2×10^−9^	1.51	0/22

(1) *Significant below 5%, ** Significant below1% with a Student test.

(2) Frequencies calculated with the first term of a Poisson law.

In general, the mutations tested had a similar effect on the yield of Lac+ and Lac− clones, compared to the wild type strain. An interesting exception is the *recD* mutation. The *recD* mutation inactivates the dsDNA exonuclease (ExoV) activity of the RecBCD complex, while maintaining its recombination potential. The overall yield of Cm^R^ Lac^+^ recombinants was increased by 4 fold, but the proportion of Cm^R^ Lac^−^ was decreased 14 fold. These two effects are those expected for the exonuclease mutant: the 4-fold overall increase in recombination is due to the higher amount of linear DNA substrate that escapes ExoV degradation. It suggests that in a RecBCD+ background, with 4 fold less recombinants compared to the *recD* mutant, only ¼ of the linear molecules resist degradation, i.e. ¾ of the linear molecules are degraded. This degradation would have been even more pronounced if pJA2 had not been protected by its two Chi sites (see Methods). Therefore in the *recD* background, pJA2 linear molecules remain undegraded, can close up and integrate by SCO (see Figure 3: increase of the right pathway). The lower yield of Lac^−^Cm^R^ is also due to an escape from ExoV-mediated degradation of the *lacZ*end region: the *lacZ* segment remains complete and gives rise more often to a Lac^+^ recombinant (Figure 3, decrease of the left pathway). Actually, sequencing the junction in the three Lac^−^ clones obtained in the *recD* mutant revealed that none of them had suffered *lacZ*end degradation. These 3 clones were not produced by HFIR either, but rather resulted from a SCO between an undeleted pJA2 and the chromosome. Their Lac^−^ phenotype was due to spontaneous mutations elsewhere in *lacZ*, either a 16 or 59 bp long deletion, or a 5 bp insertion in *lacZ*, but away from the junction (not shown). More generally, a PCR analysis of the Lac− clones obtained in the various genetic backgrounds was done, to discriminate between true HFIR events and a *lacZ* duplication (oligonucleotides j18 and j19). Results are reported in Table 5, last column. We conclude that HFIR events were not drastically enriched in any of these backgrounds, as most events observed were again *lacZ* duplications.

The most striking conclusion of this genetics study is that SCO are almost independent of both the RecFOR and the RecBCD pathway, and inhibited by RuvA.

## Discussion

During plasmid conjugation, in *E. coli*, HFIR at the *lacZ* locus occurs at a low frequency of 3×10^−10^, it is therefore not a frequent by-product of conjugation events. We can also exclude that HFIR is not detected due to the competition with a more efficient process removing the appropriate linear substrate. Indeed, the various steps of this study allow drawing a picture of the fate of linear DNA in *E. coli*: the pJA2 linear molecules are first degraded by RecBCD, and our measurements in the *recD* background suggest a frequency of degradation “D” of 75% of the molecules (this degradation would have been much higher in the absence of Chi sites). Among the molecules escaping degradation, a ligation step occurs, at a frequency “L” which we can estimate to be 10%, because the yield of recombinants with linear pJA2, in the *recD* background (2×10^−5^, Table 5), is 10% that of recombinants with circular pJA2 in the wild type background (3.3×10^−4^, Table 4). Finally, some of the circular molecules enter the chromosome by SCO, at a frequency “H” of 3×10^−4^ as deduced from the number of recombinants with circular pJA2 in the wild type background (Table 4, first lane). An even smaller fraction integrates the chromosome by illegitimate recombination (frequency I = 5×10^−7^, Figure 4 panel pJA1, value for uncut DNA). Therefore 22.5% of the linear molecules should remain linear and not degraded, providing sufficient substrate for HFIR. The remaining pJA2 molecules that do not recombine with the chromosome, both linear and circular, are probably diluted across generations, being non replicative.

A way to check whether estimations for D, L and H are correct, is to calculate the predicted frequency “F” of Lac^+^ clones obtained with pJA2 after linearization by double cutting: it should be the product of the probabilities “1-D” of not being degraded, “L” of being ligated, and “H” of recombining by SCO:
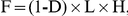
This gives F = (0.25)×(0.1)×(3×10^−4^) = 7.5×10^−6^, a value not far from the observed value of 4.2×10^−6^ (Table 5).

We also detected events resulting from a partial RecBCD-dependent degradation of linear molecules, followed by ligation and SCO (Figure 3, right panel), which led to the very same phenotype as the HFIR events, and were 10 times more frequent than HFIR. The frequency of this event (Lac^−^ Cm^R^) being 4×10^−9^, we can deduce that partial degradation occurs at a frequency D′ such that:
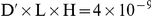
Using the values estimated for L and H, this gives D′ = 1.3×10^−4^. This illustrates the reported high processivity of RecBCD is on its dsDNA substrate [13], once loaded, it does not unload frequently.

Our initial bioinformatics observation of abundant insertions coupled with deletions of short DNA fragments (also called dimorphic loci) among *E. coli* genomes is therefore unlikely to be accounted for by HFIR as suggested initially, and these rearrangements might be formed by more complex events [7]. Visual inspection of 47 dimorphic loci revealed that in 70% of the cases, the two supposedly different variable segments detected by multiple genomes alignment had in fact some level of similarity. Homeologous recombination between distantly related DNA sequences could thus explain such situations [7]. The low efficiency of HFIR in *E. coli* during conjugation suggests that it is a process essentially restricted to naturally competent species, where the activities that process the incoming linear DNA might promote their efficient insertion coupled with a deletion in the chromosome. The observations reported in this work may even suggest that during transformation of these species, some protein might prevent the circularisation of linear double-strand DNA, to permit HFIR, or that DNA enters the chromosome in a single-strand state.

In the process of this work, we nevertheless came across a surprising observation, apparently unnoticed previously, that the exact mechanism by which SCO is taking place in *E. coli* is unknown at present. We report here that it is almost independent of both RecBCD and RecF pathways. Rather than supposing that the DNA substrate needed to initiate the recombination process is present on the incoming plasmid molecule, it may be that it is present on the recipient chromosome. For instance, a gap might be present in the process of some DNA repair (mismatch repair or nucleotide excision repair) or simply at a replication fork. Indeed, several reports point to the possible presence of RecA at stalled replication fork, with the need of a helicase such as UvrD to remove RecA from such forks [12], [14], [15]. In line with this hypothesis, we also report that SCO is increased in a *uvrD* background. However, RecA loading at the fork is thought to be *recF* dependent, an observation incompatible with the data reported here. The absence of any *recF*, *recO* or *recR* phenotype in our experimental set up remains puzzling, and suggest that RecA may use still other mediators, or none at all, to load onto certain substrates. We also report that RuvABC apparently inhibits SCO. This had already been reported in a different recombination assay based on replicative plasmids [16]. The proposed interpretation was that Ruv-mediated resolution might favour the non-cross-over products. Besides its role in resolution, RuvAB is also known to reverse some types of stalled replication forks [17]. It may be that by doing so, it removes the substrate with which circular plasmids usually interact for recombination. In conclusion, this work raises the interesting question of how SCO recombinants are produced in *E. coli*.
